# Prognostic and predictive values of the grading system of lymph node status in patients with advanced-stage gastric cancer

**DOI:** 10.3389/fonc.2023.1183784

**Published:** 2023-06-13

**Authors:** Xue-Mei Zhang, Wei-Wei Shen, Ling-Jun Song

**Affiliations:** ^1^ Pathology Center, Shanghai General Hospital, Shanghai Jiao Tong University School of Medicine, Shanghai, China; ^2^ Key Laboratory of Cell Differentiation and Apoptosis of Chinese Ministry of Education, Institutes of Medical Sciences, Shanghai Jiao Tong University School of Medicine, Shanghai, China

**Keywords:** gastric cancer, lymph node, germinal center, prognosis, chemotherapy resistance

## Abstract

**Background:**

Lymph node metastasis is one of the most important prognostic factors of gastric cancer. However, the effect of germinal centers in lymph nodes on the prognosis of patients with gastric cancer has not been reported. This study aimed to investigate the contribution of germinal center generation to prognostic parameters and clinicopathological significance in gastric cancer.

**Methods:**

We retrospectively reviewed gastric cancer patients who underwent surgery from October 2012 to June 2022. We analyzed 5484 lymph nodes (210 patients) and calculated the lymph node metastasis rate (LNMR) and the proportion of non-metastatic lymph nodes containing three or more germinal centers (NML-GCP).

**Results:**

Using a grading system that incorporated LNMR and NML-GCP. The tumors were classified into three groups based on this system, which was found to be significantly associated with prognosis. The TNM stage and grading system of lymph node status were independent risk factors for overall survival (OS) and disease-free survival (DFS). The 5-year OS rates for patients with advanced gastric cancer were 85.07% (n=50), 58.34% (n=42), and 24.44% (n=21) for Grades 1, 2, and 3, respectively (*p*<0.0001). The 5-year DFS rates were 65.32% (n=58), 40.85% (n=51), and 5.88% (n=34), respectively (*p*<0.0001). Patients with Grade 1 advanced gastric cancer had higher 5-year OS and DFS rates compared to those with Grade 2 or 3 in TNM stage II and III. Furthermore, the 5-year OS and DFS rates differed significantly among patients with different grades of advanced gastric cancer who received chemotherapy (*p*<0.0001).

**Conclusion:**

These findings suggest that the grading system may be valuable for predicting prognosis and guiding clinical management in patients with gastric cancer, and provides good prognostic stratification for OS and DFS in patients with TNM stage II and III.

## 1 Introduction

Gastric cancer is a highly malignant disease and one of the leading causes of cancer-related deaths worldwide (1, 2). Due to its insidious onset and rapid progression, most patients with gastric cancer are diagnosed at an advanced stage, which affects their prognosis. However, early-stage gastric cancer has a good prognosis, with a 5-year survival rate of over 90% (3). Treatment for advanced gastric cancer usually includes a combination of surgery, adjuvant chemoradiotherapy, molecular-targeted therapy, and immunotherapy (4). In China, chemotherapy is the conventional treatment for advanced gastric cancer, and common chemotherapeutic drugs include fluorouracil/capecitabine, taxanes (paclitaxel or docetaxel), platinum-based drugs, or a combination of these drugs.

Lymph node metastasis is a critical factor in determining the prognosis of gastric cancer (5). Germinal centers are microstructures that form within secondary lymphoid tissues in response to certain types of immunization and foreign pathogens (6). A mature germinal center consists of two compartments: a dark zone and a light zone. The relationship between the generation of germinal centers in lymph nodes and the prognosis of gastric cancer is not well understood. Therefore, this study aimed to investigate the contribution of germinal center generation to prognostic parameters and clinicopathological significance in gastric cancer. Does the production of germinal centers indicate an enhanced anti-tumor response in the body?

The study also aimed to establish a lymph node status score, analyze its relationship with clinical factors, and determine its impact on treatment. Lymph node status is an essential factor in determining the prognosis and treatment of gastric cancer patients. The number of lymph nodes involved and the extent of lymph node metastasis can influence the choice of surgical procedure and the need for adjuvant therapy.

In conclusion, the generation of germinal centers in lymph nodes may have implications for the immune response to tumors, but more research is needed to determine its role in the prognosis and treatment of gastric cancer. The establishment of a lymph node status score and its relationship with clinical factors can provide valuable information for managing and treating gastric cancer patients.

## 2 Materials and methods

### 2.1 Lymph node assessment

**Figure 1:**
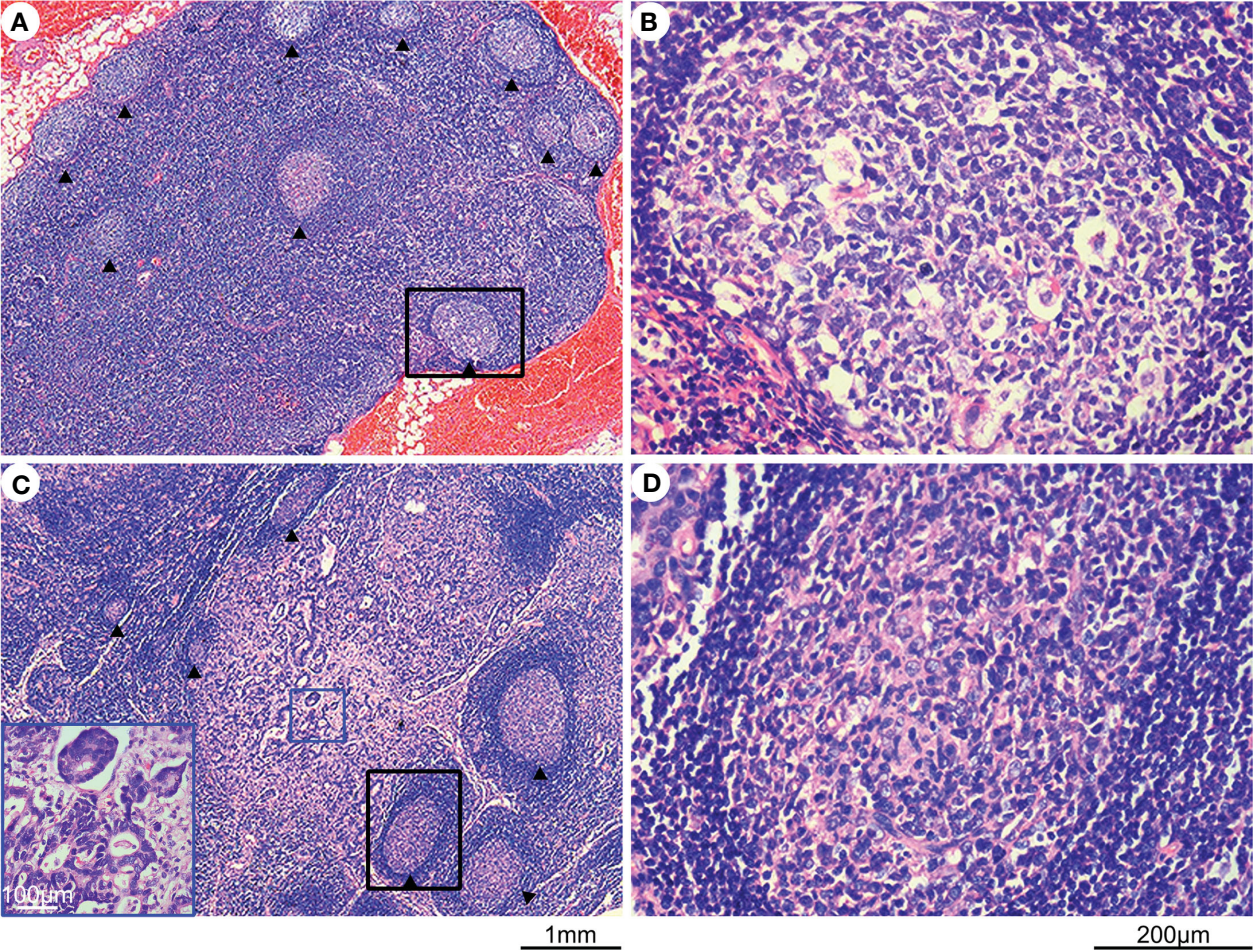
Hematoxylin and eosin (H&E) staining of lymph nodes with and without metastasis. **(A)** H&E-stained lymph node without metastasis, with germinal centers indicated by an arrow. **(B)** Enlargement of the black rectangular profile in panel **(A)**, showing a closer view of the germinal center. **(C)** H&E-stained lymph node with metastasis, with germinal centers indicated by an arrow and metastatic tumor tissues highlighted by a blue rectangle. **(D)** Enlargement of the black rectangular profile in panel **(C)** showing a closer view of the germinal center.

A total of 210 cases of gastric cancer were collected from the Shanghai General Hospital, Shanghai Jiao Tong University School of Medicine between October 2012 and June 2022. Ethical approval was obtained from an ethical review board. Tumor and lymph node specimens were fixed in 4% neutralized formaldehyde, embedded in paraffin, sectioned into 4-μm slices, and stained with hematoxylin and eosin. The staging of each gastric cancer was evaluated according to the eighth edition of the TNM staging guidelines. Two independent observers (X. -M. Zhang and L. -J. Song) evaluated all sections. The number of lymph nodes containing germinal centers was calculated (**Figures 1A–D**; median, 3; mean, 6.45; range, 0-141; the total number of lymph nodes, 5484), and the presence of ≥3 lymph follicles containing germinal centers in lymph nodes was considered positive. The positive proportion of germinal centers in lymph nodes was evaluated in each gastric cancer case, and the proportion of metastatic tumor deposits in lymph nodes was also assessed.

### 2.2 Study design and patients

We excluded 30 patients without 3-year disease-free survival (DFS) data and 12 cases with fewer than 10 lymph node evaluations, leaving 168 patients for analysis. Of these, 137 had overall survival (OS) data, with 124 receiving postoperative chemotherapy, 29 not receiving chemotherapy, and 15 with unknown treatment status.

### 2.3 Statistical analysis

We compared categorical variables using the exact chi-square test and continuous variables using the independent sample t-test, Mann-Whitney U test, and One-Way ANOVA. We used univariate and multivariate analyses to identify factors related to gastric cancer prognosis. All variables that were statistically significant in univariate analysis (*p<*0.05) were included in a multivariable Cox proportional hazards regression model (Cox regression, parameter, forward: LR). We analyzed DFS and OS using standard Kaplan–Meier analysis with a log-rank test. Statistical significance was set at a two-tailed p-value <0.05. We performed statistical analyses using SPSS, version 26.0 software (IBM Corporation, Armonk, NY, USA).

## 3 Results

### 3.1 Association between the germinal centers and metastatic tumor deposits in lymph nodes

**Figure 2:**
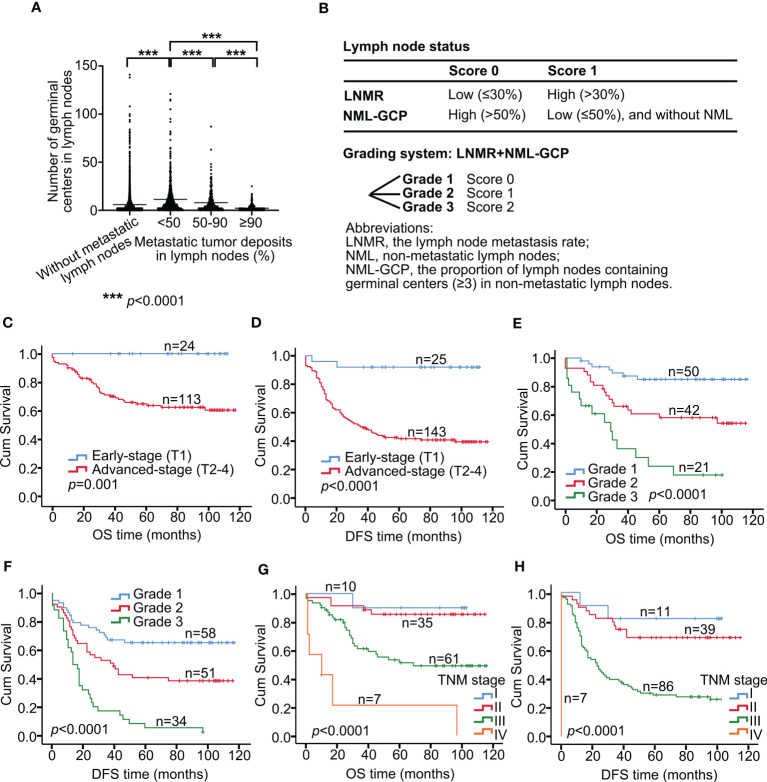
Lymph node grading system predicts survival in advanced gastric cancer. **(A)** Association between germinal centers and metastatic tumor deposits in lymph nodes. **(B)** Development of a grading system incorporating LNMR and NML-GCP. Tumors with high LNMR (>30%) were assigned a score of 1, while those with low LNMR (≤30%) were assigned a score of 0. Similarly, tumors with high NML-GCP (>50%) were assigned a score of 0, while those with low NML-GCP (≤50%) were assigned a score of 1. The scoring system for lymph node status was then graded as 0 (grade 1), 1 (grade 2), or 2 (grade 3) for each tumor. Kaplan–Meier curves show OS **(C)** and DFS **(D)** in patients with early-stage and advanced-stage (**C**, *p*=0.001; **D**, *p*<0.0001). Kaplan–Meier curves show OS **(E)** and DFS **(F)** in advanced gastric cancer patients with the Grade 1, 2, and 3 (**E**, *p*<0.0001; **F**, *p*<0.0001). Kaplan–Meier curves show OS **(G)** and DFS **(H)** in advanced gastric cancer patients with the TNM stages I, II, III, and IV (**G**, *p*<0.0001; **H**, *p*<0.0001). LNMR, the lymph node metastasis rate; NML-GCP, the proportion of lymph nodes containing germinal centers (≥3) in non-metastatic lymph nodes. OS, overall survival; DFS, disease-free survival.

In this study, the association between germinal centers and metastatic tumor deposits in lymph nodes was investigated. The authors analyzed 5484 lymph nodes, of which 1325 had metastases. The median value of germinal centers in the metastatic lymph nodes was 4, with a mean value of 8.14 and a range of 0-121. In contrast, the median value of germinal centers in 4159 non-metastatic lymph nodes was 3, with a mean value of 5.91 and a range of 0-141. The percentage of tumor deposits in metastatic lymph nodes was divided into 10 groups, and there was no difference among groups <10%, 10-20%, 20-30%, 30-40%, and 40-50%. Additionally, there was no difference among groups 50-60%, 60-70%, 70-80%, and 80-90% according to One-way ANOVA analysis (*p>*0.05, **Supplementary Table 1**). Therefore, the tumor deposits were further divided into three groups: <50%, 50-90%, and ≥90%. The authors found that the mean number of germinal centers in metastatic lymph nodes in the <50% group was 11.38, while it was 7.95 and 2.17 in the 50-90% and ≥90% groups, respectively. Significant differences were found among all three groups (*p<*0.0001, **Figure 2A**). The authors observed that as the proportion of tumor deposition increased, the number of germinal centers decreased. However, there was no significant difference in the number of germinal centers in lymph nodes with tumor deposition proportion <50%. The authors also compared the number of germinal centers in metastatic lymph nodes with tumor deposition <50% (mean value, 11.38; median value, 6) with that in the non-metastatic lymph node group (mean value, 5.91; median value, 3). The results showed a significant difference between the two groups (*p<*0.0001, **Figure 2A**), suggesting that tumors may stimulate the immune response of patients. Nonetheless, as the proportion of tumor deposition increased (≥50%), the germinal centers decreased due to tumor encroachment.

### 3.2 Clinicopathologic data

A total of 168 patients with primary gastric cancer underwent surgical resection and were included in the investigation. Male predominance was observed (male/female=120:48). The age of the patients ranged from 27 to 87 years (mean age, 61.2; median age, 61 years), and the median long diameter of the tumor was 4.5 cm (mean value, 4.58; range, 0.5–14 cm). Patients were divided into four groups based on tumor location: upper third (U, n=25), middle third (M, n=39), lower third (L, n=94), and other locations (U/M or M/L, n=10). Surgical method was categorized as total gastrectomy (n=45), proximal gastrectomy (n=7), and distal gastrectomy (n=116). The extent of lymph node dissection was classified as D0 (n=151), D1 (n=5), D1+ (n=11), or D2 (n=1) (7). According to the eighth edition of the TNM staging guidelines, the patients were divided into stages I (n=35), II (n=39), III (n=87), and IV (n=7). Among the 168 patients, 67.86% (114/168) had lymph node metastasis. The lymph node metastasis rate (LNMR) ranged from 2.86% to 100% (mean value, 33.36%; median value, 26.97%) in the 114 cases of lymph node metastasis. One case had a lymph node metastasis rate of 100%. Based on the cutoff point of 30%, the patients were divided into two groups (high and low LNMR).

The proportion of lymph nodes containing germinal centers (≥3) in non-metastatic lymph nodes (NML-GCP) was evaluated in each gastric cancer case (n=167, one case without non-metastatic lymph nodes). The median NML-GCP was 53.85% (mean value, 50.48%; range, 0-96.77%). Based on the cutoff point of 50%, the patients were divided into two groups (high and low NML-GCP).

**Table 1** summarizes the associations between LNMR, NML-GCP, and the clinicopathological features. The results indicate that LNMR was significantly associated with sex (*p=*0.017), tumor long diameter (*p<*0.0001), tumor differentiation (*p=*0.009), TNM stage (*p<*0.0001), depth of tumor invasion (*p<*0.0001), prognosis (*p<*0.0001), and recurrence and metastasis (*p<*0.0001). However, there were no significant differences between LNMR and age, tumor location, surgical method, and the extent of lymph node dissection (*p>*0.05). On the other hand, NML-GCP was significantly related to surgical method (*p=*0.045), TNM stage (*p=*0.046), depth of tumor invasion (*p=*0.002), prognosis (*p<*0.0001), and recurrence and metastasis (*p=*0.046), but there were no significant differences between NML-GCP and sex, age, tumor long diameter, tumor differentiation, tumor location, the extent of lymph node dissection, and lymph node metastasis (*p>*0.05).

**Table 1:** Relationship between LNMR, NML-GCP, and the clinicopathological features.

Clinical characteristic	LNMR (n=168)				NML-GCP (n=167)			
	No.	>30% (%)	≤30% (%)	*p* value^a^	No.	>50% (%)	≤50% (%)	*p* value^a^
Sex				**0.017**				0.792
Male	120	30 (25)	90 (75)		120	64 (53.33)	56 (46.67)	
Female	48	21 (43.75)	27 (56.25)		47	24 (51.06)	23 (48.94)	
Age (years)				0.924				0.720
≤60	80	24 (30)	56 (70)		80	41 (51.25)	39 (48.75)	
>60	88	27 (30.68)	61 (69.32)		87	47 (54.02)	40 (45.98)	
Tumor long diameter (cm)				**<0.0001**				0.273
≤4.5	92	15 (16.3)	77 (83.7)		92	52 (56.52)	40 (43.48)	
>4.5	76	36 (47.37)	40 (52.63)		75	36 (48)	39 (52)	
Tumor differentiation				**0.009**				0.107
Well-differentiated	10	2 (20)	8 (80)		10	6 (60)	4 (40)	
Moderately differentiated	48	7 (14.58)	41 (85.42)		48	31 (64.58)	17 (35.42)	
Poorly differentiated	110	42 (38.18)	68 (61.82)		109	51 (46.79)	58 (53.21)	
Tumor location				0.286				0.829
Upper third (U)	25	5 (20)	20 (80)		25	12 (48)	13 (52)	
Middle third (M)	39	14 (35.90)	25 (64.10)		39	19 (48.72)	20 (51.28)	
Lower third (L)	94	27 (28.72)	67 (71.28)		93	52 (55.91)	41 (44.09)	
Others (U/M, M/L)	10	5 (50)	5 (50)		10	5 (50)	5 (50)	
Surgical method				0.163				**0.045**
Total gastrectomy	45	16 (35.56)	29 (64.44)		45	17 (37.78)	28 (62.22)	
Proximal gastrectomy	7	0 (0)	7 (100)		7	3 (42.86)	4 (57.14)	
Distal gastrectomy	116	35 (30.17)	81 (69.83)		115	68 (59.13)	47 (40.87)	
Extent of lymph node dissection				0.279				0.580
D0	151	44 (29.14)	107 (70.86)		150	79 (52.67)	71 (47.33)	
D1	5	1 (20)	4 (80)		5	2 (40)	3 (60)	
D1+	11	5 (45.45)	6 (54.55)		11	7 (63.64)	4 (36.36)	
D2	1	1 (100)	0 (0)		1	0 (0)	1 (100)	
TNM stage				**<0.0001**				**0.046**
I	35	0 (0)	35 (100)		35	22 (62.86)	13 (37.14)	
II	39	0 (0)	39 (100)		39	26 (66.67)	13 (33.33)	
III	87	48 (55.17)	39(44.83)		86	37 (43.02)	49 (56.98)	
IV	7	3 (42.86)	4 (57.14)		7	3 (42.86)	4 (57.14)	
Depth of tumor invasion				**<0.0001**				**0.002**
T1	25	1 (4)	24 (96)		25	14 (56)	11 (44)	
T2	28	1 (3.57)	27 (96.43)		28	23 (82.14)	5 (17.86)	
T3	15	6 (40)	9 (60)		15	9 (60)	6 (40)	
T4	100	43 (43)	57 (57)		99	42 (42.42)	57 (57.58)	
Prognosis				**<0.0001**				**<0.0001**
Alive	97	13 (13.4)	84 (86.6)		97	63 (64.95)	34 (35.05)	
Dead	40	20 (50)	20 (50)		39	12 (30.77)	27 (69.23)	
Recurrence and distant metastasis				**<0.0001**				**0.046**
No	101	14 (13.86)	87 (86.14)		100	59 (59)	41 (41)	
Yes	67	37 (55.22)	30 (44.78)		67	29 (43.28)	38 (56.72)	
Lymph node metastasis								0.609
(-)					54	30 (55.56)	24 (44.44)	
(+)					113	58 (51.33)	55 (48.67)	

LNMR, the lymph node metastasis rate; NML-GCP, the proportion of lymph nodes containing germinal centers (≥3) in non-metastatic lymph nodes; No., number of cases. ^a^ Bold values indicate significance, p<0.05.

### 3.3 Association between the clinicopathological features and lymph node status

We categorized tumors based on their lymph node status using a scoring system that incorporated LNMR and NML-GCP (**Figure 2B**). Tumors with high LNMR (>30%) were assigned a score of 1, while those with low LNMR (≤30%) were assigned a score of 0. Similarly, tumors with high NML-GCP (>50%) were assigned a score of 0, while those with low NML-GCP (≤50%) were assigned a score of 1. The scoring system for lymph node status was then graded as 0 (grade 1), 1 (grade 2), or 2 (grade 3) for each tumor.

The associations between the grading of lymph node status and clinicopathological features are presented in **Table 2**. Tumors with grade 3 lymph node status were significantly associated with tumor long diameter (*p=*0.001), tumor differentiation (*p=*0.002), TNM stage (*p<*0.0001), depth of tumor invasion (*p<*0.0001), prognosis (*p<*0.0001), recurrence and metastasis (*p<*0.0001), and lymph node metastasis (*p<*0.0001). However, no significant differences were observed between the grading system and sex, age, tumor location, surgical method, or extent of lymph node dissection (*p>*0.05).

**Table 2:** Relationship between grading of lymph nodes status and clinicopathological features.

Clinical characteristic	No.	Grade 1	Grade 2	Grade 3	*p* value^a^
Sex					0.176
Male	120	54 (45)	46 (38.33)	20 (16.67)	
Female	48	17 (35.42)	17 (35.42)	14 (29.17)	
Age (years)					0.595
≤60	80	35 (43.75)	27 (33.75)	18 (22.5)	
>60	88	36 (40.91)	36 (40.91)	16 (18.18)	
Tumor long diameter (cm)					**0.001**
≤4.5	92	46 (50)	37 (40.22)	9 (9.78)	
>4.5	76	25 (32.89)	26 (34.21)	25 (32.89)	
Tumor differentiation					**0.002**
Well-differentiated	10	4 (40)	6 (60)	0 (0)	
Moderately differentiated	48	26 (54.17)	20 (41.67)	2 (4.17)	
Poorly differentiated	110	41 (37.27)	37 (33.64)	32 (29.09)	
Tumor location					0.051
Upper third (U)	25	11 (44)	10 (40)	4 (16)	
Middle third (M)	39	16 (41.03)	12 (30.77)	11 (28.21)	
Lower third (L)	94	39 (41.49)	41 (43.62)	14 (14.89)	
Others (U/M, M/L)	10	5 (50)	0 (0)	5 (50)	
Surgical method					0.091
Total gastrectomy	45	16 (35.56)	14 (31.11)	15 (33.33)	
Proximal gastrectomy	7	3 (42.86)	4 (57.14)	0 (0)	
Distal gastrectomy	116	52 (44.83)	45 (38.79)	19 (16.38)	
Extent of lymph node dissection					0.637
D0	151	65 (43.05)	56 (37.09)	30 (19.87)	
D1	5	2 (40)	2 (40)	1 (20)	
D1+	11	4 (36.36)	5 (45.45)	2 (18.18)	
D2	1	0 (0)	0 (0)	1 (100)	
TNM stage					**<0.0001**
I	35	22 (62.86)	13 (37.14)	0 (0)	
II	39	26 (66.67)	13 (33.33)	0 (0)	
III	87	20 (22.99)	36 (41.38)	31 (35.63)	
IV	7	3 (42.86)	1 (14.29)	3 (42.86)	
Depth of tumor invasion					**<0.0001**
T1	25	13 (52)	12 (48)	0 (0)	
T2	28	22 (78.57)	6 (21.43)	0 (0)	
T3	15	8 (53.33)	2 (13.33)	5 (33.33)	
T4	100	28 (28)	43 (43)	29 (29)	
Prognosis					**<0.0001**
Alive	97	56 (57.73)	35 (36.08)	6 (6.19)	
Dead	40	7 (17.5)	18 (45)	15 (37.5)	
Recurrence and distant metastasis					**<0.0001**
No	101	53 (52.48)	40 (39.6)	8 (7.92)	
Yes	67	18 (26.87)	23 (34.33)	26 (38.81)	
Lymph node metastasis					**<0.0001**
(-)	54	30 (55.56)	24 (44.44)	0 (0)	
(+)	114	41 (35.96)	39 (34.21)	34 (29.82)	

No., number of cases. ^a^ Bold values indicate significance, p<0.05.

### 3.4 Association between the clinicopathological features and prognosis

In this study, we divided patients into two groups based on the depth of tumor invasion: early stage (T1) and advanced stage (T2-4). The 5-year OS rates for gastric cancer patients with early stage, and advanced stage were 100% (n=24), and 64.20% (n=113), respectively (*p=*0.001, **Figure 2C**), whereas the 5-year DFS rates were 92% (n=25), and 42.07% (n=143), respectively (*p<*0.0001, **Figure 2D**).

Univariate analysis showed that tumor long diameter (≤4.5 vs. >4.5 cm; OS, *p=*0.035; DFS, *p=*0.031), TNM stage (I, II, III, and IV; OS, *p<*0.0001; DFS, *p<*0.0001), depth of tumor invasion (T2, T3, and T4; OS, *p=*0.001; DFS, *p<*0.0001), lymph node metastasis (negative vs. positive; OS, *p=*0.050; DFS, *p=*0.004), recurrence and distant metastasis (no vs. yes; OS, *p<*0.0001; DFS, *p<*0.0001), LNMR (≤30% vs. >30%; OS, *p<*0.0001; DFS, *p<*0.0001), NML-GCP (≤50% vs. >50%; OS, *p<*0.0001; DFS, *p<*0.0001), and grading system of lymph node status (Grade 1, 2 and 3; OS, *p<*0.0001; DFS, *p<*0.0001) significantly influenced OS and DFS in patients with advanced gastric cancers (**Table 3**).

**Table 3:** Univariate analyses for OS and DFS among patients with advanced gastric cancer.

Clinical characteristic	OS (Univariate)				DFS (Univariate)			
	No.	3-year OS	5-year OS	*P* ^a^	No.	3-year DFS	5-year DFS	*P* ^a^
Sex				0.135				0.278
Male	84	68.91%	60.48%		101	49.51%	39.08%	
Female	29	78.78%	74.63%		42	52.38%	49.62%	
Age (years)				0.078				0.104
≤60	52	77.78%	73.39%		65	53.85%	50.53%	
>60	61	66.21%	56.17%		78	47.44%	34.94%	
Tumor long diameter (cm)				**0.035**				**0.031**
≤4.5	52	82.43%	76.07%		68	55.88%	50.99%	
>4.5	61	61.87%	53.50%		75	45.33%	34.04%	
Tumor differentiation				0.120				0.093
Well-differentiated	5	80.00%	80.00%		6	66.67%	66.67%	
Moderately differentiated	36	83.15%	73.42%		42	69.05%	53.19%	
Poorly differentiated	72	64.81%	58.27%		95	41.05%	35.52%	
Tumor location				0.661				0.414
Upper third (U)	21	60.71%	54.64%		22	54.55%	44.08%	
Middle third (M)	20	79.41%	73.30%		31	45.16%	33.87%	
Lower third (L)	66	73.34%	64.62%		80	53.75%	47.25%	
Others (U/M, M/L)	6	62.50%	62.50%		10	30.00%	20.00%	
Surgical method				0.077				0.674
Total gastrectomy	36	58.74%	55.29%		42	45.24%	34.39%	
Proximal gastrectomy	5	60.00%	30.00%		5	60.00%	30.00%	
Distal gastrectomy	72	78.47%	70.63%		96	52.08%	45.59%	
Extent of lymph node dissection				0.569				0.541
D0	106	71.51%	64.86%		131	51.15%	43.01%	
D1	1	100%	100%		3	33.33%	33.33%	
D1+	6	66.67%	44.44%		8	50.00%	33.33%	
D2	0	NA	NA		1	0.00%	0.00%	
TNM stage				**<0.0001**				**<0.0001**
I	10	90.00%	90.00%		11	81.82%	81.82%	
II	35	91.43%	85.41%		39	74.36%	68.64%	
III	61	61.24%	51.29%		86	39.53%	28.76%	
IV	7	21.43%	21.43%		7	0.00%	0.00%	
Depth of tumor invasion				**0.001**				**<0.0001**
T2	27	96.30%	96.30%		28	89.29%	89.29%	
T3	5	80.00%	53.33%		15	26.67%	17.78%	
T4	81	62.16%	53.50%		100	43.00%	32.77%	
Lymph node metastasis				**0.050**				**0.004**
(-)	30	86.67%	79.86%		33	69.70%	63.36%	
(+)	83	65.66%	57.91%		110	44.55%	35.57%	
Recurrence and distant metastasis				**<0.0001**				**<0.0001**
No	78	80.77%	75.20%		78	80.77%	75.20%	
Yes	35	46.00%	33.21%		65	13.85%	3.08%	
LNMR				**<0.0001**				**<0.0001**
High (>30%)	32	42.95%	34.36%		50	26.00%	13.09%	
Low (≤30%)	81	82.23%	75.20%		93	63.44%	57.67%	
NML-GCP				**<0.0001**				**<0.0001**
High (>50%)	61	82.96%	79.25%		74	62.16%	57.73%	
Low (≤50%)	51	59.05%	48.11%		68	38.24%	26.47%	
Grading system of lymph node status				**<0.0001**				**<0.0001**
Grade 1	50	89.56%	85.07%		58	67.24%	65.32%	
Grade 2	42	66.29%	58.34%		51	52.94%	40.85%	
Grade 3	21	36.67%	24.44%		34	17.65%	5.88%	

LNMR, the lymph node metastasis rate; NML-GCP, the proportion of lymph nodes containing germinal centers (≥3) in non-metastatic lymph nodes; OS, overall survival; DFS, disease-free survival; HR, hazard ratio; 95% CI, 95% confidence interval; NA, not applicable; No., number of cases.

a Bold values indicate significance, p<0.05.

The TMN stage consists of the depth of tumor invasion, lymph node metastasis, and distant metastasis; and the grading system includes LNMR and NML-GCP, so tumor long diameter, the TMN stage, and the grading system were included in multivariate analysis. Multivariate analysis revealed that the TNM stage (OS, *p=*0.001; DFS, *p<*0.0001, **Table 4**) and grading system of lymph node status (OS, *p=*0.001; DFS, *p=*0.003) remained significantly associated with OS and DFS in the multivariate analysis model. The median follow-up times for advanced gastric cancer patients with the Grade 1, 2, and 3 were 80.5, 67.5, and 19 months, respectively. The 5-year OS rates for advanced gastric cancer patients with the Grade 1, 2, and 3 were 85.07% (n=50), 58.34% (n=42), and 24.44% (n=21), respectively (*p<*0.0001, **Figure 2E**), whereas the 5-year DFS rates were 65.32% (n=58), 40.85% (n=51), and 5.88% (n=34), respectively (*p<*0.0001, **Figure 2F**). The 5-year OS rates for patients with TNM stage I, II, III, and IV gastric cancer were 90.00% (n=10), 85.41% (n=35), 51.29% (n=61), and 21.43% (n=7), respectively (*p<*0.0001, **Figure 2G**). The corresponding 5-year DFS rates were 81.82% (n=11), 68.64% (n=39), 28.76% (n=86), and 0% (n=7), respectively (*p<*0.0001, **Figure 2H**).

**Table 4:** Multivariate Cox proportional hazard analysis for OS and DFS among patients with advanced gastric cancers.

Clinical characteristic	OS		DFS	
	HR (95% CI)	*P* ^a^	HR (95% CI)	*P* ^a^
Tumor long diameter (cm)	NA	0.523	NA	0.971
≤4.5				
>4.5				
TNM stage	3.076 (1.603-5.903)	**0.001**	3.437 (1.926-6.130)	**<0.0001**
I	1 (reference)	NA	1 (reference)	NA
II	1.442 (0.168-12.362)	0.739	1.778 (0.397-7.955)	0.452
III	3.080 (0.394-24.047)	0.283	4.003 (0.944-16.977)	0.060
IV	16.443 (1.871-144.500)	**0.012**	133.098 (18.762-944.223)	**<0.0001**
Grading system of lymph node status	2.143 (1.368-3.356)	**0.001**	1.589 (1.170-2.159)	**0.003**
Grade 1	1 (reference)	NA	1 (reference)	NA
Grade 2	2.453 (0.976-6.167)	0.056	1.607 (0.890-2.902)	0.116
Grade 3	5.699 (2.073-15.666)	**0.001**	2.935 (1.565-5.503)	**0.001**

OS, overall survival; DFS, disease-free survival; HR, hazard ratio; 95% CI, 95% confidence interval; NA, not applicable; No., number of cases.

a Bold values indicate significance, p<0.05.

**Figure 3:**
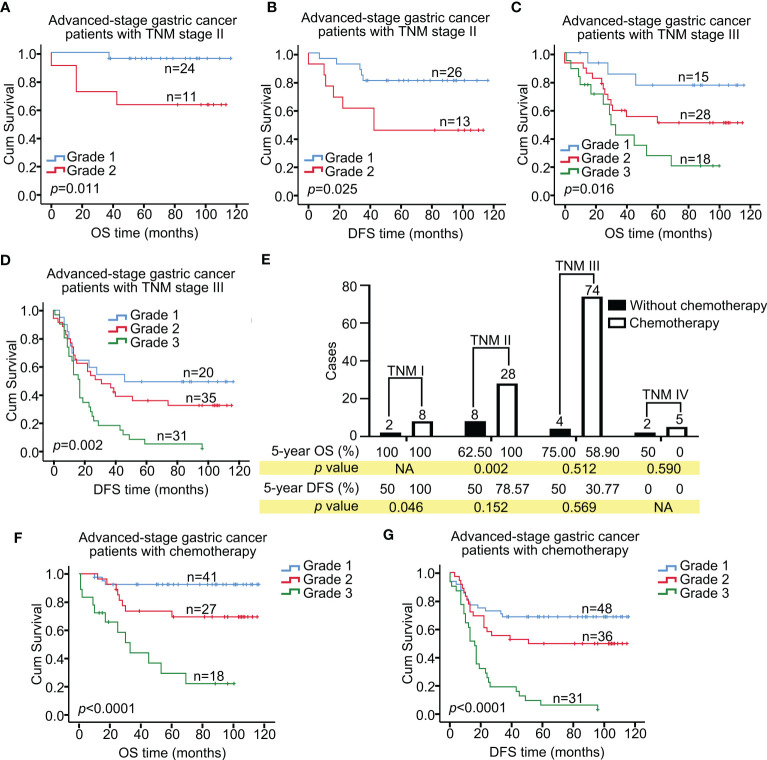
Kaplan-Meier analyses of OS and DFS in advanced-stage gastric cancer patients: impact of TNM stage, Grade, and chemotherapy. Kaplan–Meier curves show OS **(A)** and DFS **(B)** for Grade 1, and 2 advanced gastric cancer patients with TNM stage II (**A**, *p*=0.011; **B**, *p*=0.025). Kaplan–Meier curves show OS **(C)** and DFS **(D)** for Grade 1, 2, and 3 advanced gastric cancer patients with TNM stage III (**C**, *p*=0.016; **D**, *p*=0.002). **(E)** Five-year OS and DFS rates for patients in advanced-stage gastric cancer with TNM stages I, II, III, and IV, with and without chemotherapy. Kaplan–Meier curves show OS **(F)** and DFS **(G)** for Grade 1, 2, and 3 advanced gastric cancer patients with chemotherapy (**F**, *p*<0.0001; **G**, *p*<0.0001). OS, overall survival; DFS, disease-free survival.

The 5-year OS rates for Grade 1, and 2 advanced gastric cancer patients with TNM stage II were 95.83% (n=24), and 63.64% (n=11), respectively (*p=*0.011; Grade 3, n=0; **Figure 3A**), whereas the 5-year DFS rates were 80.77% (n=26), and 46.15% (n=13), respectively (*p=*0.025, **Figure 3B**). The 5-year OS rates for Grade 1, 2, and 3 advanced gastric cancer patients with TNM stage III were 77.38% (n=15), 51.31% (n=28), and 28.52% (n=18), respectively (*p=*0.016, **Figure 3C**), whereas the 5-year DFS rates were 50% (n=20), 36.73% (n=35), and 6.45% (n=31), respectively (*p=*0.002, **Figure 3D**). For advanced gastric cancer patients with TNM stage IV, the OS rates were 33.33% (1/3), 0% (1/1), and 0% (3/3) for Grade 1, 2, and 3, respectively (*p*=0.025), whereas the DFS rates were 0% (3/3), 0% (1/1), and 0% (3/3), respectively (*p*, not applicable). No differences were found between Grade 1, and 2 for OS and DFS in advanced gastric cancer patients with TNM stage I (*p>*0.05; Grade 3, n=0).

### 3.5 Association between grading system of lymph node status and chemotherapy sensitivity in patients with advanced gastric cancer

A total of 143 patients with advanced gastric cancer were included, with 115 patients receiving postoperative chemotherapy, 16 not receiving chemotherapy, and 12 having unknown treatment status. The 5-year OS rates for patients with and without chemotherapy were 72.43% (n=86) and 66% (n=15), respectively (*p=*0.386), whereas the 5-year DFS rates were 45.62% (n=115) and 43.75% (n=16), respectively (*p=*0.807). In patients with advanced gastric cancer, those with TNM stage I who received chemotherapy had a significantly higher 5-year DFS rate (100%) compared to those who did not receive chemotherapy (50%, *p*=0.046, **Figure 3E**), but there were no significant differences between the two groups in OS (*p*, not applicable). Among patients with advanced gastric cancer, those with TNM stage II who received chemotherapy had a significantly higher 5-year OS rate (100%) compared to those who did not receive chemotherapy (62.50%, *p*=0.002), but there were no significant differences between the two groups in DFS (*p*>0.05). There were no significant differences in OS and DFS between patients who did and did not receive chemotherapy for advanced gastric cancer with TNM stages III, and IV (*p>*0.05).

Furthermore, we found that the 5-year OS and DFS rates were significantly different among patients with different grades of advanced gastric cancer who received chemotherapy. The 5-year OS rates for patients with Grade 1, 2, and 3 were 92.43% (n=41), 69.35%, (n=27), and 29.18% (n=18), respectively (*p<*0.0001, **Figure 3F**), whereas the 5-year DFS rates were 68.75% (n=48), 49.85% (n=36), and 6.45% (n=31), respectively (*p*<0.0001, **Figure 3G**). These findings suggest that patients with Grade 1 advanced gastric cancer may be more sensitive to chemotherapy than those with Grade 2 or 3.

## 4 Discussion

Most patients diagnosed with gastric cancer are already at an advanced stage due to the insidious onset and rapid progression of the disease. Lymph node metastasis is a critical prognostic factor for gastric cancer (8). Currently, research on lymph nodes in gastric cancer focuses on lymph node metastasis and the rate of metastatic lymph nodes. Germinal centers are specialized microstructures that form in secondary lymphoid tissues, producing long-lived antibody-secreting plasma and memory B cells, which can provide protection against reinfection (9, 10). However, the effect of germinal centers in lymph nodes on the prognosis of patients with gastric cancer has not been reported. The authors observed that the median value of germinal centers in metastatic lymph nodes with tumor deposition less than 50% was 6 (mean value, 11.38), while the non-metastatic lymph node group had a median value of 3 (mean value, 5.91). The results showed a significant difference between the two groups (*p<*0.0001, **Figure 2A**), suggesting that tumors may stimulate the immune response of patients. However, as the proportion of tumor deposition increased (50% or more), the germinal centers decreased due to tumor encroachment.

In this study, tumors were categorized based on their lymph node status using a scoring system that incorporated LNMR and NML-GCP (**Figure 2B**). The scoring system for lymph node status was then graded as 0 (grade 1), 1 (grade 2), or 2 (grade 3) for each tumor. Multivariate analysis revealed that the TNM stage and grading system of lymph node status were independent risk factors for OS and DFS in patients with advanced gastric cancer. The median follow-up times for advanced gastric cancer patients with Grade 1, 2, and 3 were 80.5, 67.5, and 19 months, respectively. It suggest that patients with Grade 1 lymph node status have a favorable prognosis, characterized by low LNMR and high NML-GCP. The central role of CD8+ T cells in mediating antitumor immunity is well accepted (11). These cytotoxic T cells kill malignant cells by releasing inflammatory cytokines and cell lytic molecules, such as perforin and granzyme (12). Recent studies have also shown the association of B cells with anti-tumor immunity. These B cells are often organized into tertiary lymphoid structures (TLS), which are immune cell aggregates with lymph node-like features (germinal centers). TLS is proposed to establish a localized and sustained immune response, and B cells in TLS actively secrete antibodies that recognize tumor-associated antigens (13). TLS can be used to evaluate tumor immune surveillance and is an important prognostic factor for cancers (14–17). Therefore, we speculate that the activated germinal center in lymph nodes may participate in antitumor immunity and inhibit tumor progression by producing B cells.

Currently, prognosis prediction and therapeutic planning for gastric cancer patients depend on the widely used TNM system in clinical practice. The TNM system stratifies patients based on the primary tumor’s depth of invasion, the number of regional lymph nodes with metastasis, and distant metastasis (18–20). In this study, we divided patients into two groups based on the depth of tumor invasion: early stage (T1) and advanced stage (T2-4). The 5-year OS rates for early-stage and advanced-stage gastric cancer patients were significantly different, indicating that early-stage gastric cancer has a more favorable prognosis (**Figure 2C**). Moreover, we further divided advanced-stage patients into TNM stages I, II, III, and IV, which showed a significant association with OS and DFS in both univariate and multivariate analyses. The 5-year OS rates for Grade 1, and 2 advanced gastric cancer patients with TNM stage II were 95.83% (n=24), and 63.64% (n=11), respectively (*p=*0.011; Grade 3, n=0; **Figure 3A**), whereas the 5-year DFS rates were 80.77% (n=26), and 46.15% (n=13), respectively (*p=*0.025, **Figure 3B**). For advanced gastric cancer patients with TNM stage III, the 5-year OS rates for Grade 1, 2, and 3 were 77.38%, 51.31%, and 28.52%, respectively (*p=*0.016, **Figure 3C**), and the corresponding 5-year DFS rates were 50%, 36.73%, and 6.45%, respectively (*p=*0.002, **Figure 3D**). The results of this study suggest that patients with Grade 1 lymph node status have a better prognosis, as indicated by low LNMR and high NML-GCP. Moreover, the grading system for lymph node status is a reliable tool for stratifying the prognoses of both OS and DFS in advanced-stage gastric cancer patients with TNM stage II and III.

In terms of chemotherapy, TNM stage I/II gastric cancer patients had a good prognosis with chemotherapy. However, there were no significant differences between chemotherapy and prognosis in patients with TNM stage III/IV gastric cancer (**Figure 3E**).

Patients with Grade 1 advanced gastric cancer who underwent chemotherapy had higher 5-year OS and DFS rates compared to those with Grade 2 or 3, indicating that they may be more sensitive to chemotherapy (**Figures 3F, G**).

However, our study was subject to several limitations. Firstly, we lacked information on the dosage and duration of chemotherapy treatment, and the low rate of preoperative chemotherapy in our sample. Secondly, the paper does not provide any relevant data on whether the amount of germinal center-generated material is caused by pathogens. Furthermore, due to the lack of detailed information on specific lymph node grouping in some cases, the study evaluated all available lymph node sections, but did not analyze them based on lymph node grouping. In future research, we plan to investigate whether the number of germinal centers in different groups of lymph nodes varies.

In conclusion, our findings emphasize the importance of the TNM stage and grading system for lymph node status in the prognosis of advanced-stage gastric cancer. Further studies are needed to investigate the optimal dosage and duration of chemotherapy for these patients.

## 5 Conclusion

In summary, the TNM staging and lymph node grading systems are independent risk factors for OS and DFS in patients with advanced-stage gastric cancer. The lymph node grading system is a reliable predictor of both OS and DFS in patients with TNM stage II and III disease. Furthermore, patients in the Grade 1 group may benefit from chemotherapy.

## Data availability statement

Datasets for this research are available through figshare (https://figshare.com/search?q=10.6084%2Fm9.figshare.23290892) or upon request.

## Ethics statement

The studies involving human participants were reviewed and approved by Shanghai General Hospital affiliated to Shanghai Jiao Tong University. Written informed consent for participation was not required for this study in accordance with the national legislation and the institutional requirements. The statistical methods of this study were reviewed by our research support center and deemed appropriate for the purpose of this research.

## Author contributions

L-JS contributed to the study concepts, study design, data acquisition, quality control of data and algorithms, data analysis and interpretation, statistical analysis, manuscript preparation, manuscript editing, and manuscript review. X-MZ performed the data acquisition and manuscript review. W-WS contributed to the quality control of data and algorithms. All authors contributed to the article and approved the submitted version.
