# Supplementary material for: Prognostic and predictive values of the grading system of lymph node status in patients with advanced-stage gastric cancer
Source: Front Oncol. 2023 Jun 13;13:1183784. doi: 10.3389/fonc.2023.1183784 (PMC10299729; doi:10.3389/fonc.2023.1183784)
Supplement: Supplementary file 1 [file Table_1.docx]

**Supplementary Table 1. Association between the germinal centers and metastatic tumor deposits in lymph nodes**

| **Metastatic tumor deposits in lymph nodes (%)** | **Lymph node No.** | **Germinal centers** | |  | ***P* value** | | | | | | | | | |
| --- | --- | --- | --- | --- | --- | --- | --- | --- | --- | --- | --- | --- | --- | --- |
|  |  | **Mean** | **Median** |  | **<10** | **10-20** | **20-30** | **30-40** | **40-50** | **50-60** | **60-70** | **70-80** | **80-90** | **≥90** |
| **<10** | 203 | 12.34 | 6.00 |  | NA | 0.177 | 0.993 | 0.242 | 0.161 | 0.000 | 0.017 | 0.034 | 0.004 | 0.000 |
| **10-20** | 154 | 10.58 | 6.00 |  | 0.177 | NA | 0.246 | 0.998 | 0.735 | 0.022 | 0.233 | 0.251 | 0.106 | 0.000 |
| **20-30** | 109 | 12.36 | 8.00 |  | 0.993 | 0.246 | NA | 0.298 | 0.203 | 0.001 | 0.034 | 0.050 | 0.010 | 0.000 |
| **30-40** | 98 | 10.58 | 6.00 |  | 0.242 | 0.998 | 0.298 | NA | 0.754 | 0.040 | 0.277 | 0.283 | 0.145 | 0.000 |
| **40-50** | 72 | 9.99 | 6.00 |  | 0.161 | 0.735 | 0.203 | 0.754 | NA | 0.117 | 0.484 | 0.458 | 0.308 | 0.000 |
| **50-60** | 108 | 7.06 | 4.00 |  | 0.000 | 0.022 | 0.001 | 0.040 | 0.117 | NA | 0.375 | 0.535 | 0.542 | 0.000 |
| **60-70** | 88 | 8.63 | 4.00 |  | 0.017 | 0.233 | 0.034 | 0.277 | 0.484 | 0.375 | NA | 0.893 | 0.759 | 0.000 |
| **70-80** | 53 | 8.34 | 4.00 |  | 0.034 | 0.251 | 0.050 | 0.283 | 0.458 | 0.535 | 0.893 | NA | 0.901 | 0.001 |
| **80-90** | 107 | 8.08 | 4.00 |  | 0.004 | 0.106 | 0.010 | 0.145 | 0.308 | 0.542 | 0.759 | 0.901 | NA | 0.000 |
| **≥90** | 333 | 2.17 | 1.00 |  | 0.000 | 0.000 | 0.000 | 0.000 | 0.000 | 0.000 | 0.000 | 0.001 | 0.000 | NA |
| **Total** | 1325 | 8.14 | 4.00 |  |  |  |  |  |  |  |  |  |  |  |

Abbreviation: No., number of cases.
